# Experiences of and recommendations for LGBTQ+-affirming substance use services: an exploratory qualitative descriptive study with LGBTQ+ people who use opioids and other drugs

**DOI:** 10.1186/s13011-023-00581-8

**Published:** 2024-01-03

**Authors:** Margaret M. Paschen-Wolff, Avery DeSousa, Emily Allen Paine, Tonda L. Hughes, Aimee N.C. Campbell

**Affiliations:** 1 Department of Psychiatry, Columbia University Irving Medical Center and New York State Psychiatric Institute, 1051 Riverside Drive, New York, NY 10032 USA; 2 Center for Sexual and Gender Minority Health Research, Columbia University School of Nursing, 560 W. 168th Street, New York, NY 10032 USA

**Keywords:** Bisexual, Cultural sensitivity, Gay, Lesbian, Sexual minorities, Substance use disorders, Substance use treatment, Transgender

## Abstract

**Background:**

Lesbian, gay, bisexual, transgender, queer, and other LGBTQ populations (LGBTQ+; e.g., asexual individuals) have higher rates of substance use (SU) and disorders (SUD) compared to heterosexual and cisgender populations. Such disparities can be attributed to minority stress, including stigma and discrimination in healthcare settings. LGBTQ+-affirming SU treatment and related services remain limited. The purpose of this exploratory qualitative descriptive study was to characterize LGBTQ+ people’s experiences in SU services and recommendations for LGBTQ+- affirming care.

**Methods:**

We conducted demographic surveys (characterized using descriptive statistics) and individual qualitative interviews with *N* = 23 LGBTQ+ people. We employed flexible coding and a thematic analysis approach to describe participants’ experiences with stigma, discrimination, and support within SU services at the patient-, staff-, and organizational-level; and participant recommendations for how to make such services LGBTQ+-affirming. We highlighted components of minority stress and mitigators of adverse stress responses throughout our thematic analysis.

**Results:**

Patient-level experiences included bullying, name-calling, sexual harassment, and physical distancing from peers; and support via community-building with LGBTQ+ peers. Staff-level experiences included name-calling, denial of services, misgendering, lack of intervention in peer bullying, and assumptions about participants’ sexuality; and support via staff advocacy for LGBTQ+ patients, holistic treatment models, and openly LGBTQ+ staff. Organizational-level experiences included stigma in binary gendered program structures; and support from programs with gender-affirming groups and housing, and in visual cues (e.g., rainbow flags) of affirming care. Stigma and discrimination led to minority stress processes like identity concealment and stress coping responses like SU relapse; support facilitated SU treatment engagement and retention. Recommendations for LGBTQ+-affirming care included non-discrimination policies, LGBTQ+-specific programming, hiring LGBTQ+ staff, routine staff sensitivity training, and gender-inclusive program structures.

**Conclusions:**

LGBTQ+ people experience stigma and discrimination within SU services; supportive and affirming care is vital to reducing treatment barriers and promoting positive health outcomes. The current study offers concrete recommendations for how to deliver LGBTQ+-affirming care, which could reduce SU disparities and drug overdose mortality overall.

**Supplementary Information:**

The online version contains supplementary material available at 10.1186/s13011-023-00581-8.

## Background

Lesbian, gay, bisexual, transgender, queer, and other populations included in the LGBTQ community (LGBTQ+; e.g., asexual individuals) have higher rates of substance use (SU) and substance use disorders (SUD) compared to heterosexual and cisgender populations [1–9]. LGBTQ+ SU disparities include higher rates of opioid use and disorders (OUD) [6, 9, 10], as well co-use of other substances [11, 12] like stimulants [11], which together have significantly and steadily amplified drug overdose mortality in the U.S. over the past twenty-plus years [13–16]. Despite these disparities, a 2022 scoping review found limited research on opioid use and OUD among LGBTQ+ and particularly transgender populations [17]. Research has since documented higher levels of OUD [10] and opioid overdose [18] among transgender vs. cisgender individuals, and further confirmed higher rates of OUD among lesbian, gay, and bisexual (LGB) vs. heterosexual individuals [9]. SU treatment engagement and retention has the potential to dramatically reduce the risk of drug overdose mortality; deterrence of ongoing treatment engagement, including through stigma and discrimination, can be fatal in the context of the current drug overdose crisis [19].

Opioid and other SU disparities [20, 21], including unmet treatment needs [22, 23], may be understood in the context of the minority stress framework [24, 25], which suggests that LGBTQ+ people may use substances to cope with unique interpersonal and structural stressors [20, 26–28] like family rejection, internalized homophobia, anti-LGBTQ+ policies [24, 25, 29], and healthcare discrimination [2]. Although LGBTQ+ people may sometimes use substances with positive outcomes (e.g., augmentation of sexual pleasure and intimacy; mitigation of gender dysphoria by altering a sense of self) [30], SU can progress to SUD. Risk of SUD is particularly elevated in the absence of social support and affirming healthcare [24, 25, 29] and with the presence of LGBTQ+-related discrimination and stigma [20, 21, 31]. For example, a nationally representative survey of lesbian, gay, bisexual (LGB), and questioning adults found that high vs. low rates of discrimination (including in healthcare settings) doubled the odds of SUD [20]. A 2021 online survey of transgender and gender diverse individuals in the Northeast found that transgender-based discrimination predicted increased levels of past-year SUD [21].

Not only does LGBTQ+-related stigma and discrimination contribute to SUD among LGBTQ+ populations (likely exacerbated by expanding anti-LGBTQ+ legislation) [32], but it also uniquely reduces LGBTQ+ uptake of needed general health services [2, 26, 33, 34]. Research on SU treatment experiences of LGBTQ+ people, however—particularly qualitative research—remains sparse. Much of the available research is close to or over 15 years old [35] and/or from the perspective of providers [36–39]. More recent research has begun to quantitatively explore the connection between discrimination and stigma, SU treatment, and SU outcomes. For instance, the aforementioned 2021 online survey of transgender and gender diverse adults found a connection between poor treatment in healthcare settings, opioid misuse, and unfulfilled SU treatment needs, as well as a positive association between social support, reduced opioid misuse, and improved engagement in SU treatment [22].

To improve access to life-saving treatment among LGBTQ+ people, who disproportionately experience OUD and other SUD, LGBTQ+-affirming SU treatment is critical [1, 34, 39–43]. Since 2014, the U.S. Department of Health and Human Services has designated improved access to LGBTQ+-affirming care as a research priority [44]. Limited research has centered the voices of LGBTQ+ people to guide the development of affirming care. Understanding LGBTQ+ experiences with and recommendations for opioid and other SU-related services is critical to addressing SU and SUD disparities and treatment barriers, particularly amid the worsening U.S. drug overdose epidemic [13].

### Purpose

We conducted individual in-depth interviews with LGBTQ+ people who experienced OUD, other SUD, and SU treatment. Our purpose was to: (1) characterize LGBTQ+ people’s experiences of (or attitudes about) sexual orientation and gender identity (SOGI)-related discrimination and support in treatment and related services (e.g., 12-step programs; syringe exchange services); and (2) describe interviewees’ recommendations for how to make such services LGBTQ+-affirming. Due to the ongoing drug overdose crisis [13], with opioids as a driving factor in increasing overdose mortality [13, 45] and elevated rates of opioid use and OUD among LGBTQ+ populations [9, 10, 22], the study design focused on LGBTQ+ people engaged in illicit opioid use. Ultimately, all participants reported poly-SU and histories of treatment for various SUDs; therefore, this report describes experiences with and recommendations for various types of SU treatment, including for OUD.

## Methods

### Participants

The study team was based in New York; however, we recruited participants nationally via: (1) flyers posted within a LGBTQ+-focused health center (just before the COVID-19 pandemic); (2) social media posts (e.g., general posts on X (formerly Twitter), Facebook, and Instagram; posts within online queer communities); (3) an email via professional listservs to treatment providers in New York and New Jersey who shared the study information with their clients; and (4) word of mouth. Between March and October 2020, we screened 56 potential participants who contacted the study team. Of those screened, 26 were eligible and 23 completed the qualitative interview.

Eligibility criteria included: (1) age 18 or older; (2) able to speak and understand English; (3) report at least monthly illicit opioid use (i.e., other than as prescribed by a medical doctor) within the past 12 months, or up to two years prior if currently identifying as being in recovery; and (4) identify as LGBTQ+. We included participants with illicit opioid use up to two years prior to study enrollment in 2020 given that fentanyl and its analogs had already been dramatically influencing the illicit opioid supply since 2013 [46]. In addition to meeting opioid use eligibility criteria, all enrolled participants also reported poly-SU.

We enrolled participants until reaching data saturation, when the research team met to review preliminary codes and themes (one meeting after completing the first 19, and another after completing the remaining four interviews) and determined that interviews were no longer revealing new information or themes pertaining to the research questions of LGBTQ+ people’s SU treatment/services experiences and recommendations for LGBTQ+-affirming care [47]. Given the exploratory nature of the current study and the dearth of research in these areas, our interview questions were intended to elicit broad and general themes of participants’ individual treatment and services experiences and thus we intentionally sought a broad sample in terms of geography, SOGI, and treatment type. For example, the larger context of where people lived was less important to answering our research questions than people’s individual treatment experiences. Prior research suggests that when saturation goals are related to the appearance of new codes and thematic categories rather than the emergence of new theories (i.e., at an earlier phase of analysis, such as an exploratory study aimed at setting the stage for an under-researched topic), saturation may be reached at an earlier stage and with a smaller sample size [48].

### Procedures

Potential participants were instructed to contact the study team via phone, text, or email. By phone, we reviewed a study information sheet for screening, obtained verbal consent, and completed an approximately 10-minute study screener in which we also collected demographic information. Demographic information included: age in years; sexual orientation (“In terms of your sexual orientation, do your consider yourself to be [check all that apply]: lesbian, gay, bisexual, queer, pansexual, asexual, straight/heterosexual, something else [please describe]”); sex assigned at birth; gender (“How would you describe yourself now?: female or woman, male or man, genderqueer, non-binary, intersex, something else [please describe],” followed by the question, “Do you identify as transgender or as a person of a transgender experience?: [yes/no]”); past-12-month drug use “other than as prescribed to you by a medical doctor” (with a list of drugs to check off); whether the respondent identified as currently being in recovery and if so for how long; frequency of past-12-month binge drinking, opioid, and other drug use (responses of “never,” “less than monthly,” “monthly,” “weekly,” or “daily/almost daily”); whether participants had ever been in treatment for alcohol and/or drug use; whether they had ever participated in a 12-step support group; whether they had ever accessed syringe exchange services (specific treatment and service types were discussed in the qualitative interview); racial and ethnic identity; highest level of education; and the city and state from which participants were calling.

Eligible individuals then participated in one-on-one interviews via HIPAA-compliant Zoom video calls or phone after the interviewer reviewed an information sheet for the qualitative interview and the participant gave verbal consent. Participants received a copy of the study information sheet via email. Interviews lasted approximately 60 min (mean = 54 min; range: 34 to 100 min). Participants received a digital Visa gift card for $45 via text or email upon interview completion. All interviews were digitally recorded and transcribed by a professional transcription service. The New York State Psychiatric Institute Institutional Review Board approved the study.

We developed our semi-structured interview guide from extant literature on LGBTQ + barriers to accessing SU treatment services [36, 49–52] and guidelines for affirming SU-related services [1, 34, 39–43]. Interview domains included: (1) background information (i.e., pronouns; sexual orientation; gender, racial and ethnic, and other identities, and the importance of those identities for the participant; employment status; living situation; substance use timeline); (2) experiences with opioid use treatment, including types of treatment received, treatment timeline, barriers and facilitators to entering treatment, thoughts and feelings at treatment entry, and general and SOGI-related discrimination and supportive experiences while in treatment; (3) experiences with treatment for SU other than opioids; (4) for those who had never received treatment, general thoughts about SU treatment (e.g., whether participants had considered entering treatment and why might they want to in the future; concerns about accessing treatment); (5) experiences with other services and programs for opioid or other SU, including 12-step and syringe exchange; (6) for those who had never engaged in these services, general thoughts about such services; and (7) recommendations for how to make SU treatment other related services LGBTQ+-affirming. Given the dearth of research on LGBTQ+ people’s experiences in SU treatment, the current analysis focused only on SOGI-related discrimination and supportive experiences in treatment and other SU-related services, as well as recommendations for the delivery of LGBTQ+-affirming care to provide a foundation for this area of research. The other interview domains are outside the scope of this analysis.

### Positionality and reflexivity

The first and third authors conducted all interviews and generated the code book; both interviewers are white, LGBTQ+ individuals with academic and professional backgrounds in social work, public health, and/or sociology and experience working with LGBTQ+ people (personally and professionally) struggling with substance use. The second author also participated in the in-depth coding of transcripts and data analysis; this author is a Latinx, LGBTQ+ person with an academic background in psychology and professional experience in direct practice working with LGBTQ+ youth who are homeless. In terms of reflexivity and to check potential biases, these authors periodically discussed thoughts and feelings that arose during interviewing or coding related to participants’ identities (particularly SOGI, race, and ethnicity) that intersected or differed from our own. During study interviews, we further encouraged participants to relate as much detailed information as possible, consistently conveying that participants are the experts on their own identities and lived experiences, and drawing on our positionalities to convey openness and understanding of participants’ experiences while also recognizing our various differences [53].

Given that the two interviewers are white researchers with doctoral degrees working in academia, we also expressed curiosity and recognition of how participants’ identities that differed from ours—particularly race and education—may have influenced their SU treatment and service experiences. To do this, we started each interview by asking participants open-ended questions about their pronouns, sexual orientation, gender, race, ethnicity, other identities that were important to them, and what those identities meant to them, thus indicating that all identities were relevant and significant in our discussion [53]. We then asked probing follow-up questions throughout the interview about whether participants interpreted, for example, ill-treatment to be related to their salient identities, with a focus on SOGI given the focus of the study.

### Data analysis

We used descriptive statistics to characterize the demographic data. For the qualitative analysis, we employed an exploratory qualitative descriptive research design [54], which is useful for investigating topics for which there is limited prior research [54–60]. To code and analyze the data, we used flexible coding [61] paired with a thematic analysis approach [62]. The first and third study author initially read the same three transcripts, independently recorded preliminary ideas based on interview guide questions, met to discuss our notes and to develop initial broad “index codes” (i.e., based on overarching research questions) [61] from these notes, and independently coded another three transcripts using our index codes. The first and third author met a second time to discuss coding discrepancies until reaching intercoder agreement across the three transcripts (i.e., agreement on all codes within each of the three transcripts). The lead author then index-coded the remaining transcripts. After determining the focus of the current paper, the first and second author then conducted granular coding (i.e., more specific analytic codes) using the same process described above for initial index coding. We used NVivo 12 Plus to code all transcripts [63]. The first and second author then continuously organized the granular codes into categories to develop and refine themes related to the objectives of the current paper.

We describe participants’ experiences with SU treatment and related services involving SOGI-related discrimination, stigma, and support within three levels of the healthcare system: (1) interactions with peers (patient-level); (2) interactions with staff (staff-level); and (3) organizational policies and structures (organizational-level). Within these levels, we describe components of minority stress (e.g., anticipated and enacted stigma from peers and staff; identity concealment; substance use in response to stigma), as well as supportive environments, groups, and individuals who could mitigate negative impacts of minority stress [25]. We also describe participants’ recommendations for how to deliver LGBTQ+-affirming SU services. Representative quotes are included within the main text; additional and expanded quotes are in Supplementary Table 1.

## Results

### Demographic characteristics

About half (*n* = 12) of the 23 participants were residing in New York at the time of the interview; others were spread across the U.S. During the interview, many participants described treatment experiences in various states other than the state in which they were currently living, and many also described transient living situations, particularly due to COVID-19. All other demographic characteristics are reported in Table 1. The mean age was 27.5 (range: 21–38). All but two participants had at least a high school diploma or GED and most had at least some college. The sample was broadly diverse by race, ethnicity (30% Black, 17% Latinx, 13% multiracial), and SOGI. Over half identified as bisexual or queer, and nearly half identified as transgender and/or or non-binary (two of the four non-binary individuals also identified as transgender). All but two participants had participated in SU treatment and/or other related services. Close to 90% had engaged in 12-step programs like Alcoholics Anonymous (AA) and Narcotics Anonymous (NA). A little more than half had received outpatient (56.5%) and/or inpatient treatment (52.2%); a similar proportion (56.5%) had engaged in individual behavioral therapy outside of SU treatment programs. A little less than half (47.8%) had been in detox. About one in five participants (21.7%) had accessed syringe exchange services. Only two participants had been in sober living, and only one had received office-based opioid treatment. Of the 21 participants who had received SU treatment and/or other related services, all but four had engaged in inpatient and/or outpatient treatment.

About 35% of participants had received medications for opioid use disorder (MOUD). Six of the participants who were not taking MOUD had declined these medications when offered; most of these participants perceived MOUD to be worse than the opioids they were already using and were concerned that MOUD could be addictive. One participant (transgender man, age 21, white, queer) viewed Suboxone as a “very dangerous drug”. Another participant (cisgender woman, age 25, multiracial, lesbian) “*saw how Suboxone and those other [MOUD] make people…look more high to me and, I felt like I didn’t want it or trust it*.” Other than a reluctance to initiate MOUD, participants did not discuss MOUD in relation to experiences with discrimination and support within SU treatment and other services.

Over 90% of participants reported prescription opioid misuse (i.e., other than as prescribed by a medical doctor) and/or heroin use in the past 12 months, and within interviews, all of these participants described using both prescription opioids and heroin to get high. Two participants identified as being in recovery from opioid use (for 18 months and about two years respectively) at the time of the interview but reported prior weekly opioid use. Of the 21 participants who had used opioids in the past year, 17 reported daily or near-daily use. All 23 participants reported past-12-month poly-SU, including the two who identified as being in recovery from opioid use, and nearly all reported past-12-month alcohol use (including binge drinking; Table 1).

**Table 1:** Demographic characteristics of *N* = 23 LGBTQ+ participants

	Range	Mean (SD)
Age in years	21-38	27.5 (5.1)
	*n*	%
Education		
Primary school/some high school	2	8.7
High school diploma/GED	7	30.4
Some college, no degree	3	13.0
Associate degree	2	8.7
Bachelor’s degree	8	34.8
PhD, MD, or other doctoral degree	1	4.4
Race & Ethnicity		
Black/African-American, non-Hispanic	7	30.4
Latinx, any race	4	17.4
Multiracial, non-Hispanic	3	13.0
White, non-Hispanic	9	39.1
Sexual orientation		
Bisexual	5	21.7
Gay	4	17.4
Lesbian	4	17.4
Prefer not to specify	1	4.4
Pansexual	2	8.7
Queer	7	30.4
Gender identity		
Cisgender man	4	17.4
Cisgender woman	8	34.8
Non-binary (two of whom also identified as transgender)	4	17.4
Transgender man	6	26.1
Transgender woman	1	4.4
Substance use treatment and other related services experience		
Twelve-step programs (e.g., Alcoholics Anonymous, Narcotics Anonymous)	20	87.0
Outpatient treatment	13	56.5
Individual therapy for substance use (outside of a treatment program)	13	56.5
Inpatient treatment	12	52.2
Detox	11	47.8
Medications for opioid use disorder (e.g., Suboxone, methadone)	8	34.8
Syringe exchange services	5	21.7
Sober living	2	8.7
Office-based opioid treatment	1	4.3
None – i.e., no experience with substance use treatment or other related services	2	8.7
Past 12-month substance use		
Poly-substance use	23	100.0
Alcohol	22	95.65
Opioids^a^ (e.g., prescription,^b^ e.g., Oxycontin, Vicodin, Percocet], heroin, fentanyl)	21	91.30
Cannabis	14	60.87
Ecstasy	12	52.17
Hallucinogens (e.g., LSD, mushrooms)	11	47.83
Prescription^b^ sedatives (e.g., Valium, Ativan, Xanax, Klonopin)	9	39.13
Prescription^b^ stimulants (e.g., Adderall, Ritalin)	8	34.78
Poppers	6	26.09
Injection drug use (any drug)	6	26.09
Powered cocaine	5	21.74
Crack cocaine	4	17.39
Crystal methamphetamine	4	17.39
Ketamine	4	17.39
Other drugs (e.g., Gabapentin; K2 spice)	3	13.04
GHB	1	4.35
Past 12-month substance use frequency		
Binge alcohol use^c^	22	
*Daily/almost daily*	10	45.45
*Weekly*	7	31.82
*Monthly*	0	0.00
*Less than monthly*	5	22.73
Opioid use	21	
*Daily/almost daily*	17	80.95
*Weekly*	3	13.64
*Monthly*	1	4.55
*Less than monthly*	0	0.00

*NOTE LGBTQ+ *Lesbian, gay, bisexual, transgender, queer, and other populations within the LGBTQ community (e.g., asexual individuals)

^a^Two participants were in opioid use recovery at the time of the interview but reported prior weekly opioid use

^b^Used other than as prescribed by a doctor, e.g., higher quantity or frequency than prescribed, someone else’s prescription

^c^Assigned female at birth = 4 or more drinks in one sitting; assigned male at birth = 5 or more drinks in one sitting

### Interactions with peers (patient-level)

#### Discrimination and stigma– overt

Multiple participants reported overt—i.e., direct, enacted—discrimination and stigma from peers. Such instances of overt discrimination and stigma included name-calling and homophobic slurs, being misgendered (e.g., referred to with the wrong pronouns), bullying, and harassment. For example, one participant (transgender man, age 23, Black, prefer not to specify sexual orientation) had been called a “he/she and had chips thrown” at him in AA; another (cisgender man, age 33, Black, bisexual) had been ridiculed at inpatient graduation for kissing and hugging his boyfriend and was subsequently outed on Facebook. The latter participant reported that instances of SOGI-related bullying from peers led to relapse on two separate occasions.

Multiple participants, most of whom were trans, reported receiving threats of physical violence or sexual harassment from peers in treatment settings. One cisgender gay man’s (age 29, white) roommate in an inpatient program left a letter on his bed threatening to lock him in his room at night and force him to perform sexual favors. Another recounted being sexualized by his peers both at an inpatient program and a sober house:

*“It was at that weird place in between my transition where you really can’t tell. That made them uncomfortable because they can’t label me. Or it would be the other end of the spectrum where they would sexualize and fetishize the fact you couldn’t tell… I had a guy make me super uncomfortable because he asked me to have sex with him… Also, at my first sober house… The guys there really sexualized me when they found out that I was into men… They definitely took that as an invite to flirt with me and be openly sexual towards me.”* (transgender man, age 23, white, queer).

#### Discrimination and stigma– indirect

Several participants had also experienced indirect discrimination and stigma from other clients, which we defined as participants’ perception of stigmatization based their being LGBTQ+. In some instances, this involved visible discomfort or stares from peers that participants viewed as reactions to their gender and/or sexuality. One person noticed peers “scooting away” (cisgender man, age 33, Black, bisexual) from him in an AA meeting at the sight of his painted nails. One cisgender woman (age 21, Black, queer) described feeling ignored in AA and NA, both due to her sexuality and her race:

*“I’m a fat, black, queer girl, sitting with a bunch of other 18-, 20-year-old white girls that were doing heroin and stuff, and I would try to talk about my experiences, because honestly they were either similar or worse to them, and it was just like no one seemed to even pay attention to what I was saying.”*

#### Support– shared life experiences and community-building

Several participants found support and value in “having some sort of shared experience” (as a non-binary and transgender, 29-year-old, Black, queer participant specifically noted) with LGBTQ+ peers in one-on-one interactions and group counseling settings. Only one participant (cisgender woman, age 25, multiracial, lesbian) had attended an LGBTQ+-specific treatment program; in this outpatient program, educational and social programming like movie nights and dance parties, as well as documentary viewings and workshops on LGBTQ+ rights made her feel “comfortable and accepted.” Several other interviewees attended LGBTQ+-specific support groups within general treatment programs, as well as general LGBTQ+ and transgender-specific 12-step support group meetings (e.g., AA). One transgender man (age 21, white, queer) said of his first LGBTQ+ AA meeting:

*I showed up and there were people wearing drag. There were people wearing leather. Some people were there with their partners and it just seemed like every preconception that I had about AA and God and stuff like had just been shattered at that point*.

Participants felt affirmed by LGBTQ+ peers who could understand their experiences with SOGI-related discrimination and identity development, and with whom they could center and celebrate LGBTQ+ identities and connect over community norms. For instance, one participant (cisgender woman, age 31, Black, lesbian) noted:*“[My LGBTQ+ peers and I] would have a text group and people would text you every morning…positive things that had to do with the LGBT community…We all needed that.*”

### Interactions with staff (staff-level)

#### Discrimination and stigma– overt

About half the participants also experienced overt discrimination from staff while seeking or receiving treatment. For example, two participants stated that providers called them homophobic slurs, one of whom (cisgender man, age 33, Black, bisexual) also reported two instances of being denied services, once when a nurse in his detox program refused to assist him, and later while calling local clinics to see if they were LGBTQ+-affirming:

“… *As soon as I told three of those [programs] that I was openly bisexual, they hung up. I’ve had one tell me to search other…alternative rehab… It felt like what was the point of even trying to get off of drugs if you’re going to have that kind of ignorance from people?*”

A few people reported being misgendered or dead-named (i.e., called by their birth name) by staff. A staff member accidentally outed one transgender participant by telling him in the presence of other clients that:

“…*it was time to go upstairs…they put me up on the women’s floor because [the staff] claimed it was the only place…that they had a…single room available. And then everybody kind of looked at me like … why do you go up there?… I programmed with the men downstairs. But unless [the other clients] asked, they wouldn’t even know that [I was transgender]*” (transgender man, age 21, white, queer).

Further, a cisgender, bisexual woman (age 36, white) reported an instance of a medical provider sexually harassing her by asking invasive questions (e.g., “*How did it feel when you were with her?*”) exclusively about her female partners.

#### Discrimination and stigma– indirect

Several people reported less overt instances of discrimination and stigma from staff, such as non-verbal cues or tacit behaviors that participants perceived as related to their LGBTQ+ identities. For example, one transgender man (age 38, Latinx, queer) stated: “*I felt at times, like especially when I was in the state-funded places, I felt people uncomfortable with me because they perceived me to be gay*.” In some instances, participants encountered therapists who appeared to be generally unwelcoming based on participants’ LGBTQ+ identities without being explicitly discriminatory.

#### Discrimination and stigma – limited staff intervention

Even when staff were not directly stigmatizing toward LGBTQ+ clients, several participants described lack of staff intervention as a key contributing factor to a stigmatizing environment. In these instances, peers enacted stigma via discriminatory language, threats of violence, and bullying and staff amplified such stigma by failing to name or disrupt it, or by actively dismissing or minimizing participants’ concerns. For example, one participant stated that after he was physically assaulted by another patient because of his sexual orientation, the inpatient program director encouraged him to “… consider choosing a different facility” (cisgender man, age 29, white, gay) instead of taking steps to protect the participant and disciplining the abusive patient. Another participant reported to staff that a patient had sexually propositioned him. When he asked if that patient could be moved to another unit within the inpatient program, staff responded, “*You’re both being discharged in a couple days, it will be fine*” (transgender man, age 23, white, queer).

In other instances, lack of staff intervention meant that participants carried the burden of addressing discrimination and stigma from other clients, rather than staff members taking on that responsibility. This was evident in one participant’s description of a session with their intensive outpatient counselor:

*“I was expressing my frustration of being misgendered. People would call me [by the correct name] but then they would use ‘she/her’ pronoun stuff. And I just didn’t feel [that my counselor] was very supportive of that. She was just kind of like, ‘You should bring it up’ … And I guess that part of it might have been that she was trying to empower me or something, but it just felt a little lonely.”* (transgender man, age 28, Latinx, gay).

#### Discrimination and stigma– assumptions about sexuality, gender, and race

In addition to lacking staff support, a few respondents also encountered assumptions from staff about their sexual identity or behavior, such as staff presuming clients’ sexual identity labels based on their reported sexual history rather than asking clients to self-label, or staff profiling LGBTQ+ clients as more promiscuous than their non-heterosexual peers. For example, when one participant (transgender man, age 38, Latinx, queer) told his provider that he was married to a woman and attracted to men, the provider “… *[implied] that [I was] a cheater or that I’m lying to myself or someone else, when clearly, I’m being completely out with everything*.”

Two participants’ providers suggested that participants would want to sleep with their peers based on their sexual identity. In one case, a provider assumed that a participant and his close friend were in a relationship “… *because two people are LGBT, they obviously have to be interested in each other. So, they were trying to separate us and put us as far away from each other as they could*” (cisgender man, age 29, white, gay). Another respondent (transgender man, age 23, white, queer) noted: “… *it was heavily insinuated by staff that I couldn’t be left alone with any - I had to have eyes on me, like one on one because in my health chart it says, ‘high risk sexual behavior*’”.

Participants also described staff—specifically individual therapists outside of treatment programs—who made assumptions about clients based on their gender, racial, and/or sexual identities, which led to preconceived ideas about participants’ SU histories and treatment needs. In some instances, staff made generalizations about trans clients, thus potentially overlooking participants’ unique experiences and missing opportunities to adequately address underlying causes of addiction. One participant (transgender man, age 28, Latinx, gay) reported having a therapist who

“… *sometimes [will] assume about my experience based on another client of his who’s trans …. there is some commonality to our experiences, but we’re all individual people too*. *We have different perspectives and opinions and beliefs and things that are unique to us*.”

Another participant (transgender man, age 38, Latinx, queer) cited his transgender identity as a positive aspect of his life and felt “frustrated” with past therapists who assumed a connection between SOGI-related trauma, LGBTQ identity, and SU. The participant recounted an experience where after his therapist learned that he was trans, “*not only was [my being trans] the only thing she could talk about, she was convinced that the reason I didn’t talk about it was because it’s the root of all of my problems*.”

Another participant (cisgender woman, age 21, Black, queer) discussed staff assumptions about her race and sexuality that also led to inadequate treatment. The participant described racist experiences where, due to providers’ preconceived notions about what “hard drug users” look like, providers viewed her drug use as “experimenting” rather than as a serious problem warranting treatment. One experience with an individual therapist who identified as gay had been especially disappointing, particularly because the participant had anticipated that an LGBTQ+ therapist would show more curiosity about and openness to learning about her life. Instead, the participant again seemed to feel dismissed and disillusioned, and still had not received much-needed comprehensive or supportive treatment that could have addressed the root causes of her addiction:

“*Recently, I think, was about a year ago, I started going and seeing a therapist who was a gay white man. So, I thought that would be a little more helpful…But it was still the same thing where I would say something that, I guess, that people wouldn’t align with the way I look [as a ‘fat, Black, queer woman’]…he just didn’t believe…or listen to…my hard drug use. I would tell him about…experiences I’ve had, mostly relating to drugs, because that’s kind of what I wanted to talk to him about. He didn’t really respond…I couldn’t really get into depth…he would always steer the conversation to some other thing that I was talking about that I didn’t really want to get into… and [discussions of my drug use] would just be completely pushed under the rug for the rest of the meeting*.”*”* (cisgender woman, age 21, Black, queer).

#### Discrimination and stigma– absence of direct SOGI discussions

Participants also sometimes related that an overall absence of direct SOGI discussions in treatment created an unsupportive and unwelcoming environment, and one in which important connections between LBGTQ+ identities and addiction continued to be ignored. For example, one participant (cisgender man, age 29, white, gay) said that while discussions about SOGI were not outwardly discouraged within his inpatient program, *“[he] definitely didn’t feel comfortable talking about it*,” which meant that he was unable to openly discuss relationships and self-esteem issues related to his sexuality that had contributed to his SUD. Participants also expressed frustration when treatment seemed to focus only on their SU struggles without a more in-depth examination of the interplay between having a marginalized identity and a SUD. As one person stated about their experience in inpatient treatment:

“*It was just hard to find a place to even talk about [sexual orientation and gender identity]; because I mean, it does play into who I am obviously…it also influences a lot of the reasons why I started using…it was…hard to talk about that because I was almost always the only LGBT person there at all. So, it felt like I could either go somewhere and talk about my gender identity and sexuality, or I could go somewhere and talk about my substance abuse. Never felt like I could talk about both of those things. So that was tough*.” (*transgender man, age 28, Latinx, gay*).

#### Discrimination and stigma – identity concealment response

Due to either experiencing or anticipating SOGI-related discrimination and stigma, a few participants reported concealing their identities in SU treatment settings. After a negative first experience seeking treatment led to a relapse, one participant (cisgender man, age 33, Black, bisexual) attempted to conceal his orientation during his next treatment experience: “*Maybe if I hide [my bisexuality] for as long as I can, I can get some kind of treatment for a while and maybe… I won’t relapse*.” Another respondent (cisgender woman, age 25, multiracial, lesbian) reported that although staff in her inpatient program did not say anything outwardly discriminatory, the fact that the environment was not explicitly welcoming to LGBTQ+ people (e.g., no LGBTQ+ representation in their reading materials) meant that she “couldn’t really be [herself]” within the program.

#### Support – staff as advocates for LGBTQ+ clients in otherwise stigmatizing environments

While participants often described staff members who reinforced and perpetuated a stigmatizing environment by failing to intervene in instances of peer-generated discrimination and stigma, more than half the participants also encountered staff who actively interceded to support clients in the face of both staff- and peer-based mistreatment like transphobia and bi[sexuality]phobia. Even in environments that were generally stigmatizing and inhospitable to LGBTQ+ clients, having at least one staff member to serve as a champion of LGBTQ+ inclusivity provided participants with a sense of comfort and safety and potentially mitigated feelings of isolation and fear.

For example, one person (cisgender man, age 33, Black, bisexual) discussed a nurse in his detox program who briefed the rest of the clinical team on his genderfluidity and bisexuality, which helped assuage staff nervousness about how to approach the participant and led to more respectful interactions with staff: “*And after [the nurse] said that, the nurses who had just come in, ‘How are you feeling today? Are you okay, sir, ma’am?’ Or, they would ask me, you know, ‘What do I want to be called?*’” Another participant (cisgender man, age 29, white, gay) noted that “*the trauma counselor…was helping me stay away from a client who was discriminating and kept me closer to [another client] that I connected with and was able to talk to*.” A third participant (transgender man, age 33, Black, prefer not to specify sexual orientation) recalled an inpatient counselor who allowed him to use her personal restroom after other patients were upset that he was using the men’s room.

#### Support – LGBTQ+-identifying and allied providers

A few participants identified providers who were openly LGBTQ+ or well-versed in LGBTQ+ issues as important sources of support who made participants comfortable being open about their own identity in treatment:

*“…[The outpatient program counselor] opened up with jokes about being a lesbian and it just made me right then feel that I don’t have to hide who I am. There are people like me. I’m not weird. I’m not funny. I’m not whatever people think I am, and I think that just was very supportive*” (cisgender woman, age 25, multiracial, lesbian).

Others appreciated when their providers understood the nuances of LGBTQ+ identities, without clients having to offer further explanation, such as the fact that sexual identity and gender identity are separate constructs, and the gender of one’s sexual partners should not be assumed based on their sexual orientation. For example, one bisexual participant (non-binary, age 31, multiracial) described a counselor who “*understood exactly what I meant*” when the participant described having a girlfriend and also flirting with a man.

Several participants also valued LGBTQ+-identified and allied providers who recognized the importance of treatment fostering patients’ exploration of the connection between addiction, SOGI-related trauma, and LGBTQ+ identities. Participants appreciated when providers understood that elements of SOGI-related minority stress (e.g., SOGI-based discrimination and stigma; identity concealment) as well as general stressors (e.g., childhood traumas unrelated to SOGI) contributed to addiction as self-medication. For example, one participant noted that she knows “*a lot of people like me have gone through like, you know…trauma when they were younger or even, you know, older or whatever. And I think that it’s important for [SU treatment providers] to kind of specialize in that…*” (cisgender woman, age 26, white, lesbian). For other participants, SU was connected to greater self-acceptance rather than traumatic experiences, alluding to the fact that SU can occur in social settings with other LGBTQ+ individuals. For example, another participant noted that “…*my gender played a huge role in my substance abuse… like I started using more after I had kind of come out and accepted myself*” (transgender man, age 21, white, queer).

### Experiences with organizational policies and structures (Organizational-Level)

#### Discrimination and stigma – gendered program structures

Participants also described experiences with discrimination and stigma at the institutional level. A few participants commented on the binary, gendered nature of treatment programs across the board, from AA meetings to sober houses to inpatient programs:

“*There are definitely gendered [AA] meetings, as well as all sober houses are gender segregated. There’s no trans or queer sober houses. They are all either men or women. I’ve always been in men’s houses, and I’ve always been the only trans person*” (transgender man, age 23, white, queer).

One non-binary and transgender participant (age 29, Black, queer), spoke about programs that will “only accept men patients or women patients.” Two other participants reported being assigned to a room according to their sex assigned at birth rather than their gender identity.

#### Support – gender-affirming program structures

By contrast, a few participants recalled being assigned to rooms that aligned with their gender or were otherwise provided with the option. One participant (transgender man, age 21, white, queer) in an outpatient program that had opened gender-inclusive housing noted that the program asked “*where [he] was most comfortable being housed*” and appreciated that “*my gender and sexuality…wasn’t really a focus…I like just being able to be integrated in with everybody else*.” This participant was also the only person who explicitly mentioned a SOGI-related non-discrimination policy within a treatment program. A few other participants also described intake processes that were particularly inclusive of LGBTQ+ people, such as staff explaining the need for questions about sex assigned at birth and including expansive and open-ended response options for sexual identity on intake forms.

#### Support – affirming treatment environment

While several participants experienced supportive policies and procedures, only three noted visual signifiers of an LGBTQ+-affirming environment. These included LGBTQ+-specific signage, rainbow flags, supportive messages, and drawings created by the clients themselves. Such visible cues signaled to participants that programs actively welcomed LGBTQ+ people by validating their existence. Physical indicators of affirming care also helped to assuage participants’ concerns about anticipated stigma by showing upfront that SOGI are important components of people’s overall identity. As one cisgender woman (age 25, multiracial, lesbian) put it, seeing rainbow flags posted within a treatment program “is about acknowledgement”.

Several participants also appreciated programs and services that took a non-judgmental, harm reduction approach. For one participant, this was the case with an individual therapist who took a patient-centered approach by working with the participant on their own SU goals rather than pushing an abstinence-only approach. As a result, the participant felt motivated to remain in treatment and that the therapist valued the client’s perspective:

“*That’s probably the first therapist that I could stick with. And he can tell things about me before I even say them; but he obviously waits for me to say them because he wants to hear it from me which I appreciate that level of attention where I know that he’s paying attention to me and picking up on things and kind of knows my patterns of whatever I’m up to…And I feel like he doesn’t judge me for my drug use. He doesn’t force me to quit…I also really appreciate that he doesn’t push the total sobriety in anything on me. Because I guess I drink every once in a while and I’ll smoke weed every once in a while like not daily; not to the point where it’s like a problem. And I appreciate that he kind of understands that and this is cool with that*.” (transgender man, age 28, Latinx, gay).

Although only about 20% of participants had accessed syringe exchange programs, those who did found them to be particularly affirming in their non-judgmental attitude. Syringe exchange programs prioritized clients’ safety above all else, had a “no questions asked” attitude, and were particularly welcoming environments for transgender people. For example, the same participant who had discussed a positive experience with their individual therapist above described their experiences with syringe exchange programs where they had been both a client and an employee:

*“They truly don’t care who you are, your background, anything like that. They just want to keep you safe and that’s it…the one here especially is really good about trans issues because they also do a lot of outreach, and there are a lot of trans women involved in that. So, they’re really, really good about being respectful of people’s genders…”* (transgender man, age 28, Latinx, gay).

Although most participants described specific experiences with SOGI-related discrimination and stigma with SU treatment settings, six participants noted that they had never encountered SOGI-related mistreatment.

### Recommendations for LGBTQ+-affirming SU treatment and services

Based on their lived experiences with addiction and SU treatment and services, participants made recommendations for how to make services LGBTQ+-affirming. Table 2 includes an outline of these recommendations. Additional representative quotes are presented in Supplementary Table 1.

**Table 2:** Recommendations from *N* = 23 LGBTQ+ people with lived experience on the provision of LGBTQ+-affirming SU treatment and services

Policies	• Develop and clearly document SOGI-related non-discrimination policies, including guidelines on disciplinary actions for staff and peers who mistreat LGBTQ+ clients with tiered responses based on severity of mistreatment (e.g., name-calling vs. sexual assault). • Develop policies on the routinization of peers sharing names and pronouns as part of introductions. • Include LGBTQ+ people in the development of non-discrimination and staff vetting policies by forming community advisory boards and meeting with LGBTQ+ people with lived experiences in addiction and SU treatment and other services.
LGBTQ+-Specific Services	• Where possible, create LGBTQ+-only SU treatment programs. If not possible, offer LGBTQ+-specific groups within larger SU treatment programs and ensure that programs are broadly inclusive of LGBTQ+ clients beyond specific groups.
Staff Hiring	• Where possible in the context of anti-LGBTQ+ legislation, hire openly LGBTQ+ (or explicitly LGBTQ+-allied) staff at all levels, from support staff to behavioral health providers to medical doctors. • Vet staff during hiring and onboarding processes for LGBTQ+-affirming views and practices.
Intake Forms and Processes	• Intake forms should include questions about the name participants go by, pronouns, gender identity, and sexual orientation, with options for open-ended responses as well as pre-written response options beyond “male” and “female”; “straight”, “lesbian”, “gay”, and “bisexual”. • Responses to SOGI intake questions should be voluntary, recognizing the potentially sensitive nature of such questions.
Provision of Affirming Treatment	• Staff should share their pronouns and ask clients to do so within group and individual treatment sessions. Sharing and asking about pronouns should be a routine component of care. • Staff should guide patients in exploring the connection between addiction, SOGI-related trauma, discrimination, and stigma, while also recognizing that identifying as LGBTQ+ does not always lead to trauma or negative health outcomes. • Staff should view LGBTQ+ clients holistically (including accounting for unique experiences based on racial, ethnic, and other salient identities) rather than only focusing on SOGI.
Staff Training	• Staff should receive LGBTQ+ sensitivity training that accounts for fear and anxiety about working with LGBTQ+ clients. Training content should guide staff in how to avoid making SOGI-related assumptions about clients based on the gender of clients’ partners, how to respectfully ask clients about SOGI, education on SOGI-related language and terminology (what does the LGBTQ+ acronym stand for; slang terms), information about LGBTQ+ communities (e.g., chosen family; importance of LGBTQ+-affirming social spaces), and guidelines for working with transgender and gender diverse clients (e.g., normalize sharing and asking about pronouns and correct names). • Staff should receive training in how to intervene when LGBTQ+ clients experience discrimination, stigma, or aggression from other clients. • Staff training should use an intersectional lens to teach staff how to effectively work with clients with intersecting marginalized identities (such as LGBTQ+ people of color). • Trainings should occur regularly (e.g., at least once a year) and should be interactive and individualized based on staff role (e.g., medical doctors, mental health providers, receptionists). • People who identify as LGBTQ+ should deliver staff trainings.
Visual Cues of an Affirming Environment	• Display rainbow flags; LGBTQ+-specific brochures, signs, and banners (e.g., posters with LGBTQ+ couples and transgender and non-binary people related to SU treatment with motivational messages); and videos with scenarios about SU that include racially diverse LGBTQ+ characters. • Ensure that programs are affirming beyond visual cues (e.g., educating the public on LGBTQ+ issues, hosting LGBTQ+ events, and connecting transgender people with affirming services such as hormone replacement therapy and name change clinics).
Gender-Affirming Program Structures	• Ensure that programs are safe spaces for transgender and non-binary people, e.g., by offering single-stall gender-neutral bathrooms or by allowing people to use the bathroom aligned with their gender; and by offering gender-neutral living arrangements or housing people based on their gender.

*NOTES LGBTQ+* Lesbian, gay, bisexual, transgender, queer, and other populations within the LGBTQ community (e.g., asexual individuals), *SOGI* Sexual orientation and gender identity, *SU* Substance use

#### Recommended policies

Participants recommended that SU treatment programs have clearly documented non-discrimination policies to address SOGI-related discrimination and stigma from staff and peers. Policies should include formal guidelines on disciplinary actions for staff and peers who mistreat LGBTQ+ clients, such as tiered responses based on the severity of the mistreatment (e.g., name-calling vs. threats of sexual assault). Two participants (two cisgender gay men, one age 22 and Black and the other age 29 and white) recommended that LGBTQ+ people “have a voice” in the development of non-discrimination and staff vetting policies within SU treatment programs by meeting with program staff and directors to provide input. Some participants noted that non-discrimination policies should specifically address gender-affirming care, including guidelines on the use of clients’ correct names and pronouns and relaxing binary gendered dress code policies. Other participants thought programs might be required to ask about sex assigned at birth and names on documents for legal and health insurance reasons but suggested that programs develop policies and procedures for how to sensitively explain this requirement to clients.

A couple participants noted that many SU treatment programs do not admit openly LGBTQ+ patients in the first place, so focusing on non-discrimination policies may not be the best starting point for promoting LGBTQ+-affirming care. One participant (cisgender woman, age 21, Black, queer) stated that *“…it doesn’t matter what the policy is in your program if [LGBTQ+ clients are] not in it*.”

#### Recommendations for LGBTQ+-specific services

Drawing on their positive experiences with LGBTQ+ peers, several participants suggested that LGBTQ+-only treatment programs “*could be very helpful in bringing a community together and getting people some help that maybe they wouldn’t get in another program*” (cisgender man, age 29, white, gay). Others recommended that programs for the general population continue to offer LGBTQ+-specific groups. Several other participants expressed ambivalence. On the one hand, having broadly inclusive programs with LGBTQ+ clients integrated into the general patient population could decrease othering of and stigma toward LGBTQ+ people. On the other hand, LGBTQ+-only programs and groups could create a sense of safety.

#### Recommendations for staff hiring

Participants also recommended that programs vet staff for LGBTQ+-affirming views and practices and where possible, promote the hiring of openly LGBTQ+ staff at all levels, from support staff to behavioral health providers to medical doctors. As one participant (non-binary, age 29, Black, queer) stated: “*…if the place is going to say that they’re going to cater to LGBTQ people, then the people that are working there [should] actually reflect that*.”

#### Recommendations for intake forms and processes

Many participants recommended that intake forms include questions about their correct name, pronouns, and gender identity, especially because clients’ current name might differ from the name on their legal documents. Many participants also suggested that rather than having check boxes with pre-written SOGI responses, forms should instead “*have lines to write on…and I understand it’s harder for data collection. But someone else can figure out how to make that work… I think that the more you can let people self-identify, self-disclose, actually the more, better information you get*” (transgender man, age 38, Latinx, queer).

For programs that do include check boxes, several participants recommended having response options beyond “male” and “female”, or “straight”, “lesbian”, “gay”, and “bisexual” since some participants may not use any of these more binary, rigid labels. Having more expansive options could not only make LGBTQ+ people feel more welcome but could also help ease the stress of initiating SU treatment. On the other hand, some participants suggested that sharing one’s SOGI at intake should be voluntary due to the sensitive nature of such questions. These participants talked about the need for balance between asking intake questions that demonstrate a program’s comfort with discussing SOGI and avoiding questions that could feel irrelevant to the treatment or overly personal:

“*I would say, [sexuality] definitely shouldn’t be a required question… It should be something that somebody voluntarily answers… But I’m not sure. I think that sometimes when I see the sexuality portion of a form, I kind of wonder why if it’s not that relevant, but I would also want to feel comfortable knowing that if I were to discuss anything about my sexuality that I wouldn’t be judged for it. So I don’t know how to meet that middle ground*.” (*transgender man, age 21, white, gay*).

#### Recommendations for the provision of affirming treatment

Participants made recommendations for providers to deliver LGBTQ+-affirming treatment in both group and individual settings, including routinely sharing their own pronouns and encouraging clients to do so as well. Participants noted that routinization of these processes could help mitigate misgendering and dead-naming from staff and peers, set a tone of inclusivity for both cisgender and transgender clients, and reduce stigma toward transgender, non-binary, and other gender-diverse clients.

As part of affirming care, participants also recommended that providers ask about and explore SOGI beyond the intake process, and should seek to understand clients holistically rather than focusing only on their gender and sexuality. Several participants noted that treatment should foster patients’ exploration of the potential connection between addiction, LGBTQ+ identities, and SOGI-related discrimination and stigma by welcoming participants to discuss their SOGI in treatment rather than avoiding or glossing over those discussions. For example, one participant noted:

*“[Providers] would just need to know that [my queerness is] a big part of who I am. And my thought process that goes into me using [drugs] in the first place has a lot to do with who I am. So just knowing or giving me space to talk about it, it would be the most helpful*.” (*cisgender woman, age 21, Black, queer*).

#### Recommendations for staff training

Nearly all participants recommended that staff receive sensitivity training around working with LGBTQ+ clients, speculating that staff may be anxious about discussing SOGI for fear of inadvertently making offensive statements. Training would thus need to account for and address staff members’ fear and anxiety. Recommended training content included how to avoid making SOGI-related assumptions based on the gender of clients’ partners, how to respectfully ask clients about SOGI, education on SOGI-related language and terminology (e.g., what does the LGBTQ+ acronym stand for; slang terms), and information about LGBTQ+ communities (e.g., chosen families; the importance of LGBTQ+-affirming social spaces). Nearly half the participants suggested that staff receive specific training on working with transgender and gender diverse clients (e.g., normalize sharing and asking about pronouns and correct names). Additionally, participants suggested that staff receive guidance on how to correct instances of misgendering without further “alienating” clients:

“*I would say [staff trainings are] one of the most important things. Having people who are queer competent…so they know how to refer to someone and how to correct themselves. That’s one of the biggest things, when I correct someone on my pronouns, I don’t want them to be like, ‘OMG I’m so sorry’. Then I feel like I have to calm them down and say it’s okay, when that’s really not my job… They should be trained to be like okay, I will adjust my language and leave it at that… It won’t happen again… Just to make sure the queer people don’t feel more alienated than they already do*.” (transgender man, age 23, white).

A few participants also indicated a need for staff training on how to intervene when LGBTQ+ clients experience discrimination, stigma, or aggression from other clients. For example, staff should receive guidance on whether to step in when clients use the wrong pronouns for other clients, and on how to address sexual harassment of LGBTQ+ clients so that LGBTQ+ people don’t feel like they are “*kind of pushed aside because the staff is afraid of handling it*” (transgender man, age 21, white). Several participants highlighted that staff should be trained to consider clients’ intersecting marginalized identities (such as LGBTQ+ people of color) and cultural backgrounds and how experiences related to those identities may be interconnected with addiction. Several participants similarly noted that staff should receive training on how LGBTQ+ people have unique experiences with discrimination and stigma (e.g., minority stress), including direct harassment from other people as well as discriminatory policies and laws. One participant (cisgender woman, age 31, white) suggested that not only should staff receive training in these areas, but SU treatment programs should also actively show support for LGBTQ+ communities by participating in LGBTQ+-supportive rallies and LGBTQ+ pride marches.

Finally, several participants gave recommendations on the format and frequency of staff training, from once a year to once a month. Other participants suggested that training should be interactive and potentially spaced out over a few days. Two participants noted that training format, frequency and comprehensiveness should depend on the staff member’s role and level of contact with patients (e.g., trainings might be different for a driver vs. a behavioral health provider). A few participants stated that people who identify as LGBTQ+, such as those who had struggled with SUD, should deliver staff trainings.

#### Recommendations for visual cues of an affirming environment

Nearly half the participants recommended that programs have visual cues in the physical environment to signal that programs are welcoming to LGBTQ+ people. For example, many participants noted that having a rainbow flag somewhere within the organization would assuage staff anxiety about raising SOGI-related topics in the treatment setting. Several participants recommended that programs hang posters of LGBTQ+ couples and transgender and non-binary people related to SU treatment with uplifting messages. Two participants proposed that programs show videos with scenarios about substance use that include racially diverse LGBTQ+ characters. Some participants also suggested that more discreet signifiers like videos, brochures, and small rainbow flags would be meaningful signifiers of an LGBTQ+-affirming environment, even if the program was not LGBTQ+-specific.

One participant (cisgender man, age 29, white, gay), however, noted that simply hanging a rainbow flag doesn’t indicate whether an organization integrates LGBTQ+-affirming practices throughout their program:

“*…this place I went to did have a rainbow flag hanging up to I guess state that [they were LGBTQ+-affirming], but didn’t necessarily make me feel more comfortable there. Like you could say, you support this, but it doesn’t necessarily mean that there’s any follow through*.”

Some participants suggested concrete steps that programs could take to be truly LGBTQ+-affirming, such as educating the public on LGBTQ+ issues, hosting LGBTQ+ events, and connecting transgender people with affirming services such as hormone replacement therapy and name change clinics.

#### Recommendations for structuring programs to be gender-affirming

In addition to recommendations for visual cues of LGBTQ+-affirming spaces, several participants also had suggestions for how spaces should be structured to affirm clients’ gender. Some participants acknowledged the nuances in creating safe spaces for transgender people without making them feel excluded. Some felt that people should be encouraged to use “*whatever [gender-segregated] bathroom you’re comfortable using*” and should be housed based on gender or in gender-neutral spaces, rather than having separate bathrooms and living spaces for LGBTQ+ people. As one transgender man (age 28, Latinx, gay) summed it up:

…*it’s hard because you don’t want to be singled out as trans person but also you want to make sure that you feel safe. Because I don’t know that I would want to room with a cis[gender] man either. So maybe like having individual rooms that LGBT people can maybe opt into; because I could also see a cis[gender] gay man not wanting to room necessarily with a straight guy depending on who that is…It’s very easy to make gender neutral bathrooms*.

## Discussion

This qualitative study describes the lived experiences of 23 racially diverse LGBTQ+ people in OUD and SU treatment and other related services. To our knowledge, this is one of the first studies to examine SU treatment experiences from the perspective of LGBTQ+ people. Most participants in the present study had experienced some form of SOGI-related discrimination or stigma in SU treatment and other services, and most had also received SOGI-related support at multiple levels. Where discrimination in healthcare can lead to SU [20, 21], supportive environments can alleviate the negative effects of minority stress experiences and reduce adverse outcomes [25] like SU, relapse and overdose [19]. Based on these lived experiences, participants made recommendations for SU treatment programs and services to reduce discrimination and stigma and increase support for LGBTQ+ clients. The current study suggests a need for SU treatment programs and related services to adopt policies and procedures such as those recommended here, with input from LGBTQ+ clients themselves. Findings also indicate important areas for future research, including understanding power dynamics between SU treatment staff and clients; exploring LGBTQ+ SU treatment clients’ experiences with intersectional discrimination and stigma; and developing and evaluating implementation strategies for LGBTQ+ affirming SU programming.

Most participants in the current study had engaged in 12-step programs, and more than half had received inpatient, outpatient, and/or individual behavioral health treatment; however, only a little more than a third had received MOUD. Several participants expressed wariness and distrust about the effectiveness of MOUD, with some viewing such medications as dangerous or harmful. This represents an area of important future research, particularly given that MOUD are considered life-saving drugs and the gold standard of OUD treatment [64]. Future studies on MOUD among LGBTQ+ people should also examine differences in acceptability and uptake by SOGI as well as intersecting identities like race, ethnicity, and age.

### SU treatment/services experiences: patient-level

Discriminatory experiences with peers included bullying, name-calling, sexual harassment, threats of physical violence, visible discomfort, and physical distancing. These findings echo those from a 2015 qualitative study of transgender individuals’ experiences with inpatient SU treatment [65]. Stigma from peers led to relapse for one participant, demonstrating a stress coping response as described within the minority stress framework [25]. Peer support included discussing subjects like shared experiences with SU, LGBTQ+-related discrimination, and LGBTQ+ identities both one-on-one and in group settings. Such support engendered a sense of community. Prior research affirms the value of peer support in SU treatment [66, 67]. For example, a 2019 systematic review found that engagement with supportive peers as part of SU treatment was associated with lower rates of SU relapse, more positive interactions with treatment providers, ongoing treatment engagement, and increased satisfaction with treatment [67]. A 2020 literature review found that peer support not only increased positive SU treatment outcomes for clients receiving such support, but also improved self-esteem and feelings of empowerment among peer workers [68]. Additionally, peer support is one of the five pillars of trauma-informed care, and thus has the potential to address LGBTQ+-related trauma within addiction treatment and create a sense of safety for LGBTQ+ clients [69].

The minority stress framework also suggests that feeling connected to an LGBTQ+ community can help mitigate the negative impact of discriminatory experiences such as LGBTQ+-related trauma [25]. LGBTQ+-specific groups and community events within SU treatment are also crucial to improving treatment outcomes [70]; however, barriers to providing and accessing such services include limited funding, not enough LGBTQ+ clients to form groups [39], (with low enrollment possibly due to enacted or anticipated discrimination) [33, 34], and inaccurate advertising of specialized LGBTQ+ SU programming [64]. Future funding should be allocated to support LGBTQ+-specific SU treatment and to more accurately identify existing SU treatment programs tailored to LGBTQ+ communities in publicly-available SU treatment directories [64].

### SU treatment/services experiences: staff-level

Like their experiences with peers, participants also reported overt, direct discrimination from staff such as SOGI-related name-calling, dead-naming, and misgendering, as well as denial of services. Participants further contended with general coldness from providers that seemed to be related to participants’ LGBTQ+ identities. Stigma from providers led to denial of services, SOGI concealment, and SU relapse in some cases. Previous research has shown that provider-based stigma presents barriers to general healthcare [21, 31, 34]. In a survey of U.S. transmasculine adults, having been refused medical care based on transgender identity predicted reduced odds of obtaining future care [31]. LGBTQ+ people have also cited anticipated stigma from providers without LGBTQ+-affirming training as a primary obstacle to utilizing care [34]. The findings from our study also echo earlier research [65, 71], suggesting that LGBTQ+ discrimination and stigma in SU treatment have not abated over the past twenty years despite federal efforts to develop guidelines on LGBTQ+ affirming care [70]. For example, research from the early 2000s revealed that providers in SU treatment settings have endorsed stigmatizing and biased attitudes toward LGBTQ+ people [36–38] and have only occasionally provided LGBTQ+-affirming care [38]. Additionally, the previously mentioned 2015 qualitative study of transgender people’s experiences in SU treatment found that participants encountered name-calling and misunderstandings about their gender on the part of staff [65].

Participants in the current study not only described overt discrimination from SU treatment staff and providers and its negative consequences, but also highlighted the absence of direct support as equally upsetting. This included a lack of staff intervention that left clients to shoulder the burden of addressing peer-based stigma. The theme of a paucity of staff intervention represents a fruitful area for future research, in which power dynamics between clients and patients could be explored to better understand how such dynamics influence the overall climate of the SU treatment setting. Particularly given that staff are in positions of power, their failure to intervene in instances of LGBTQ+-related discrimination and stigma has the potential to create hostile and unsafe environments where LGBTQ+ people become doubly isolated by both stigma from peers and lack of support from those in charge.

Relatedly, the omission of direct SOGI discussions in treatment can perpetuate feelings of isolation and disillusionment among LGBTQ+ clients. Participants also discussed frustrations with providers who made assumptions and generalizations about their gender, sexuality, and race as further contributing to disappointingly inadequate treatment. Prior literature has found that provider assumptions about SOGI reduce client trust and lead to missed opportunities for shared collaborative patient-provider decision-making about general healthcare [72], suggesting that breaks in trust similarly limit patient-centered SU treatment for LGBTQ+ clients.

Providers who were champions of LGBTQ+ support and sensitive to the nuances of LGBTQ+ identities, however, created a sense of safety and security for LGBTQ+ participants in otherwise hostile environments. Previous research has highlighted the importance of reducing discrimination and stigma from providers in general healthcare settings to reduce SU and resultant SUD as stress coping responses [20, 31, 73]. Participants in our study also expressed comfort and security with LGBTQ+-identifying and/or allied providers with whom they could be themselves and who did not require additional explanation or education about LGBTQ+ identities on the part of the clients. These findings further contribute to previous qualitative research demonstrating that LGBTQ+-identified or actively LGBTQ+-welcoming providers helped alleviate LGB clients’ concerns about sexuality-based stigma in general healthcare [74, 75]. Minimizing provider-based discrimination [31, 34] and hiring LGBTQ+-affirming providers—whether LGBTQ+-identifying or strong allies [34, 74] —can also promote SU treatment initiation and improve retention among LGBTQ+ people.

### SU treatment/services experiences: organizational-level

At the organizational level, participants felt that explicitly welcoming visual cues like rainbow flags and LGBTQ+-specific imagery would be important; however, participants cited enactment of supportive policies and structures such as gender-affirming care as more crucial than physical indicators of an affirming environment. Participant described feeling stigmatized within treatment and 12-step programs structured around binary gender identities and feeling supported from other programs that offered gender-affirming and inclusive groups and housing. Such support improved a sense of belonging and affirmation with SU treatment. Prior research has similarly stressed that gender-affirming SU treatment is crucial for improving treatment engagement among transgender clients [71, 76]. Research has also found that gender-affirming general healthcare is linked to improved mental health outcomes such as lower odds of depression and suicidality [77], which may also contribute to improved SU outcomes.

### Recommendations for LGBTQ+-affirming SU treatment and services

Based on their lived experiences with SU treatment and other services, participants’ key recommendations for LGBTQ+-affirming care included: (1) the development and enforcement of non-discrimination policies; 2) the provision of LGBTQ+-specific programming within treatment; (3) the hiring of staff with explicitly LGBTQ+-affirming views and practices, as well as openly LGBTQ+ staff (which may depend on staff comfort level in disclosing SOGI in the context of rising anti-LGBTQ+ laws and policies) [32]; (4) the inclusion of comprehensive and open-ended SOGI questions on intake forms, with responses being voluntary; (5) the delivery of LGBTQ+ sensitivity training to staff at all levels, including how to avoid making SOGI-related assumptions, how to provide holistic care that accounts for intersectional identities, and how to provide gender-affirming care; (6) the inclusion of explicit visual cues of an LGBTQ+-affirming environment; and (7) the provision of gender-affirming program structures, such as bathrooms and housing that affirm participants’ gender and welcome gender diversity.

Participant recommendations in the current study reaffirm the recommendations in the existing literature from the perspective of LGBTQ+ people with lived experience in SU treatment and addiction. For example, SAMHA’s 2012 guidelines on providing SU treatment for LGBTQ+ clients recommend that programs create a welcoming environment with physical indicators of LGBTQ+ inclusivity like rainbow flags, highlight the importance of staff training for promoting LGBTQ+-affirming care, and remind providers to treat patients holistically and account for intersectional and other marginalized identities [70]. Trainings for providers should therefore use an intersectional lens that highlights the diversity of LGBTQ+ communities, as well as how intersecting marginalized identities may compound SOGI minority stress responses like SU [78, 79]. Previous research also echoes current participants’ recommendations that providers should avoid pathologizing or narrowly focusing on LGBTQ+ identities, and that intake forms should include open-ended SOGI questions to foster LGBTQ+-affirming interactions throughout treatment and to avoid pitfalls like assuming clients’ family structures [52]. To ensure that LGBTQ+ people are actively included in developing and guiding SU treatment policies and procedures, SU treatment programs and services could develop patient or community advisory boards comprised of LGBTQ+ people with lived SU experience. Additional research could also further explore how to link organizational structures that set the stage for LGBTQ+-affirming SU treatment and the delivery of such treatment from staff members.

### Limitations

The current study is not without limitations. First, findings may be weakened by the fact that some participants were recounting experiences from several years prior to the interview. Second, although nearly half of participants were in various states, just over 50% were in New York state at the time of the interview, which may indicate a biased perspective on SU treatment experiences given New York public accommodation laws protect against discrimination and service denial on the basis of SOGI [80]. Still, many participants recounted treatment experiences in various states and may not have received treatment in the state where they were residing during the study.

Third, despite our recruitment efforts, only one participant identified as a transgender women; thus, our findings do not represent the experiences of transgender women. Also, the oldest participant was 38, and the mean age was 28, thus limiting our ability to report on SU treatment experiences of older LGBTQ+ adults. Older adults may have been underrepresented in the current study given that much of the recruitment took place via social media, a venue that typically attracts adolescents and young adults [81]. These limitations point to important areas for future research—for example, the experiences of transgender women and older LGBTQ+ adults within SU treatment.

Data for this study were collected in the early months of the COVID-19 pandemic, when there were extensive disruptions in SU treatment and other related services, as well as rising rates of SU among LGBTQ+ populations [82, 83]. Thus, the timing of the data collection could have influenced the study findings given that the additional stress of COVID-19 could have amplified participants’ existing experiences with LGBTQ+-specific stress.

Finally, the current study lacked an intersectional analysis of discrimination and stigma, including differences in outcomes by race, ethnicity, age, and SOGI. As described above, we opened the interview by asking participants to discuss their racial, ethnic, and other identities they felt were important, and what those identities meant for them. We also asked participants to describe experiences of discrimination and stigma more generally in addition to LGBTQ+-specific discrimination, with probing follow-up questions based on participants’ self-described salient identities; however, these questions did not elicit any conversations about substance use stigma or misogyny (beyond misogyny connected to SOGI) and brought forth only limited discussions of racism. The absence of these discussions may have resulted from our recruitment and interview focus on LGBTQ+-related discrimination and stigma. Individuals with intersecting marginalized identities (e.g., LGBTQ+ people of color) face multiple systems of oppression (e.g., racism and transphobia) [84] that can heighten minority stress and its sequalae, including SUD. Further research designed to generate knowledge on the racialized experiences of LGBTQ+ people and SU treatment is warranted.

## Conclusions

The current study demonstrates that LGBTQ+ people continue to experience discrimination and stigma within SU services at multiple levels, including from peers, providers, and organizational structures; yet many LGBTQ+ individuals also experience support from the same sources. Such discrimination can exacerbate minority stress processes such as identity concealment, and stress coping responses like SU relapse, while support can assuage negative outcomes of minority stress and facilitate treatment engagement and retention. Based on their experiences with SU programming, the participants in the current study recommended a range of strategies to promote affirming care, including non-discrimination policies, LGBTQ+-specific and gender-affirming programming, rigorous staff training, and direct LGBTQ+ client involvement in SU treatment planning and policy making. This study reiterates the importance of the decade-old SAMHSA guidelines on LGBTQ+-affirming SU treatment from the perspective of LGBTQ+ people in their own voices. Findings from this study may also contribute to the refinement of such guidelines, given that in the past decade LGBTQ+ people (along with the general population) have experienced a global pandemic, rising overdose mortality [13], and increasingly anti-LGBTQ+ laws and policies [32]. SU treatment programs should consider adopting these recommendations to ensure that LGBTQ+ people receive support and affirmation, which could close gaps in SU treatment access, reduce risk of drug overdoses, and promote overall health of LGBTQ+ people.

### Supplementary Information

**Additional file 1:** **Supplementary Table 1.** Additional and expanded quotes about LGBTQ+ people’s experiences with and recommendations for providing LGBTQ+-affirming substance use treatment and other services (*N*=23).

## Data Availability

The datasets generated and analyzed during the current study are not publicly available given the sensitive nature of the material (e.g., sexual orientation, gender identity, illicit substance use) but are available from the corresponding author on reasonable request.

## Abbreviations

AA: Alcoholics Anonymous
LGB: Lesbian, gay, and bisexual
LGBTQ+: Lesbian, gay, bisexual, transgender, queer, and other populations within the LGBTQ community (e.g., asexual individuals)
NA: Narcotics Anonymous
OUD: Opioid use disorder
SOGI: Sexual orientation and gender identity
SAMHSA: Substance Abuse and Mental Health Services Administration
SU: Substance use
SUD: Substance use disorders
