# Supplementary material for: Experiences of and recommendations for LGBTQ+-affirming substance use services: an exploratory qualitative descriptive study with LGBTQ+ people who use opioids and other drugs
Source: Subst Abuse Treat Prev Policy. 2024 Jan 3;19:2. doi: 10.1186/s13011-023-00581-8 (PMC10765665; doi:10.1186/s13011-023-00581-8)
Supplement: Supplementary file 1 — Additional file 1: Supplementary Table 1. Additional and expanded quotes about LGBTQ+ people’s experiences with and recommendations for providing LGBTQ+-affirming substance use treatment and other services (N=23). [file 13011_2023_581_MOESM1_ESM.docx]

**Supplementary Table 1. Additional and expanded quotes about LGBTQ+ people’s experiences with and recommendations for providing LGBTQ+-affirming substance use treatment and other services (N=23)**

| **Substance Use Treatment and Services Experiences: Interactions with Peers (Patient-Level)** | |
| --- | --- |
| Discrimination & Stigma from Peers: Overt | “I had my ride with my boyfriend at the time and when he picked me up, I hadn’t seen him in like a week… So, of course, he gives me a hug and a kiss and I don’t care. I kiss him in front of my parents and my kids. So, it doesn’t really bother me. And then everybody was just pointing and taking pictures… it didn’t really bother me until I was tagged in a picture on Facebook from somebody from the group. And then I just lost it… they ridiculed me. They tagged me. They put this picture on Facebook and was like, guess what? Guess who… came out of the closet?” (*cisgender man, age 33, Black, bisexual*)  “I had a roommate…who wrote on a letter and left it on my bed that he was at night going to lock the door to prevent anyone from coming in or going out and he wanted me to perform sexual favors to him…The next instance, a gentleman physically touched me…After shouting that he doesn’t like homos and he physically put his hands on me…” (*cisgender man, age 29, white, gay*)  “I had a guy [within inpatient treatment] call me a faggot once and want to fight me…I’m involved in some 12-step groups on Facebook that are like…non-stop faggot this, faggot that.” (*transgender man, age 38, Latinx, queer*) |
| Discrimination & Stigma from Peers: Indirect | “November of 2014, I started going [to] AA classes and I lasted…until my 90 chip. And then I had my nails done so the day before I didn’t think to take my nails off. I was and I, you know, you’re just thinking I just went because it was my day to go, and I was trying. And when I got my 90-day chip and when everybody, they stood to give you a little claps, and everybody seen my nails. It just – everybody just stood looking at me weird and scooting away from me. I was so embarrassed, and I couldn’t – at first, I couldn’t figure out why everybody was staring at me until I looked down at my hands and I was just like oh.” (*cisgender man, age 33, Black, bisexual*)  “I feel like there were people looking at me, but like, but that’s no different I guess than everyday life.” (*cisgender man, age 29, white, gay*) |
| Support from Peers: Shared Life Experiences & Community-Building | “I would say [that I felt supported by] my peers. The ones that were transgender like me and going through transitioning…We talked about our own challenges growing up and what we felt and how the world understands us, so we definitely made our own connection and bond.” (*transgender man, age 23, Black, prefer not to specify sexual orientation* )  “The only helpful thing was that you get to talk to other people who had potentially been where you are at… There was actually one dude [in NA] who was gay… I think in the [NA] meeting he had talked about he was gay and how he had grew up in religion and it was just tough doing that. And I think afterwards I told him myself, ‘You know, hey, I’m bi, so I can you know kind of get on board with what you are saying.’…He sends me emails that says you know, just motivational shit… and um, it turns out that that was actually the most useful resource for me.” (*cisgender man, age 25, white, bisexual*)  “My parents really lucked out in finding this [exclusively LGBTQ+ SU treatment] place… They would just have…a rainbow shirt day …every Friday, so I think that was more so like made us feel comfortable and accepted. And like we would have like movie night where everybody comes together and it’ll be, sometimes they made it a dance, LGBT discrimination or things like that like documentaries and things.” (*cisgender woman, age 25, multiracial, lesbian*)  “Where it’s because like I said my counselor is gay and we would do like a LGBT like group because we do like a secondary group…It was really cool…we just kind of talked about, you know, being discriminated against and just like the issues that we face.” (*cisgender woman, age 26, white, lesbian*) |
| **Substance Use Treatment and Services Experiences: Interactions with Staff (Staff-Level)** | |
| Discrimination & Stigma from Staff: Overt Discrimination/Stigma | “I had one [nurse] come in. I had my nails done and I think, I believe my boyfriend at the time had brought my wig in and I had it on. And he walked me and seen it and just walked back out and asked to be replaced with another nurse.” (*cisgender man, age 33, Black, bisexual*)  “Oh, I had a crisis – we had the counselor, um, it was – we got into a real big fight. Um, an argument. She was disrespectful. Um, you know, calling me a dyke…I wanted to see a therapist at a certain time and [the counselor] wouldn’t let me…I know she was talking about [me] – because I was the only person in that group or individual in that group that dressed – you know, um, sometimes I would dress feminine and sometimes I wouldn’t.” (*cisgender woman, age 30, Latinx, pansexual*)  “So, first I was transitioned at that point socially. I hadn’t started taking testosterone yet. They wouldn’t put me in the men’s side, which they kept saying was for safety, which I understand, but the way they handled it was so horrifying. [The staff] kept calling me a girl and a woman and being – ‘Okay girls, all right women’ to all of us. I was, ‘Hello, I look like a boy and I sound like a boy and I am a boy.’” (*transgender man, age 23, white, queer*) |
| Discrimination & Stigma from Staff: Indirect Discrimination/Stigma | “Um, I wouldn’t say disrespected, not discriminated, by my sexual orientation…I guess, it was disrespect because I felt that you know, some of [the staff] wasn’t as friendly, you go there in the morning and you know, they would be like, ‘Oh just put your stuff down to sit’. It was not like [a] greeting. There was no really follow-ups after the group or if I ever wanted to speak to my counselor, I was always getting brushed off.” (*cisgender woman, age 30, Latinx, pansexual*) |
| Discrimination & Stigma from Staff: Limited Staff Intervention | “The [inpatient] program wasn’t a warm like welcome and then I heard a client [in an AA-type meeting] say, ‘Oh they let dykes in’. So I heard like – I heard like you know negative remarks… a counselor was right there and heard and never said anything.” (*cisgender woman, age 31, Black, lesbian*)  “A gentleman physically touched me…After shouting that he doesn’t like homos and he physically put his hands on me… the director of the program spoke to me and I didn’t like, she’s like, ‘Oh, well, maybe you should consider choosing a different facility.’ Which I felt like, like I know that’s an option of mine, but to have her like say that to me, it felt like she was saying that I should choose somewhere different, so I didn’t feel like I was being supported in all this.” (*cisgender man, age 29, white, gay*) |
| Discrimination & Stigma from Staff: Assumptions from Staff about Sexuality, Gender, and Race | “Um, like [the staff] didn’t understand…like how can a person sleep with somebody that’s trans and sleep with somebody that’s male or sleep with somebody that’s female, altogether in one. They didn’t understand that. They were just like, ‘Oh well then you should be labeled as bisexual or like you know, things like that. They need to understand like no, that’s not what it is, and you can’t - you can’t label a person.” (*cisgender woman, age 30, Latinx, pansexual*)  “There was one staff member who…said something, and it was like, really kind of subversive. It wasn’t very, like overt, about my lifestyle or something. And like, I knew that like, I knew he didn’t know I was trans. But like, I’m pretty open about being queer or whatever. And I felt like he was like making a comment about that. And I was just like super mad about it…he was saying something about like where I’m pretty sure he was getting at was because I admit freely to being married to a woman but like being interested in men, also. And he made some snide comment about that. About like, I need to be more truthful or honest. Like he’s instigating that I’m like a cheater or that I’m lying to myself or someone else, when like clearly, I’m being completely out with everything.” (*transgender man, age 38, Latinx, queer*)  “There was one person that I happened to really get along with who also happens to be LGBT as well, which made it easier for me cause there were certain things that were easier to talk about with this individual. And the staff, I guess, assumed that we had to be hooking up or in a relationship because two people are LGBT, they obviously have to be interested in each other. So, they were trying to separate us and put us as far away from each other as they could. So, I felt that I was being discriminated against for that reason.” (*cisgender man, age 29, white, gay*)  “I have been frustrated with individual therapists at times who get hung up on certain things. I had one therapist years ago who like when I was in my drinking phase, this was right before I went to the first really good [treatment] place, like I didn’t go in there—I didn’t even tell her I’m trans, because I don’t feel like it has to do with anything. Like it’s one of the few things in my life that has been consistently good and has gone well. So, like I didn’t ever bother telling her, because I didn’t feel like it was relevant to anything. But then when she found out, not only was it the only thing she could talk about, she was convinced that the reason I didn’t talk about it was because it’s the root of all of my problems.” (*transgender man, age 38, Latinx, queer*) |
| Discrimination & Stigma from Staff: Absence of SOGI discussions | “It would have been nice…if there was some sort of emphasis on [sexual and gender] identity, you know what I mean? Because this affected how I, you know, obtained drugs. This affected how people viewed me as an addict. And I was basically hiding [my identity], you know. And if…I was provoked or had been asked, I mean, maybe, you know, I would’ve said something about this sooner…There was no question of like identity or like sexual interests or anything like that.” (*non-binary and transgender, age 24, white, queer*)  “[Sexual orientation] just [haven’t] come up, or it wasn’t like a topic that we were discussing deeply. [Therapists] mostly just like listened to what I was saying [about my sexual orientation] and then didn’t really have any response about it… It is frustrating.” (*cisgender woman, age 21, Black, queer*) |
| Discrimination & Stigma from Staff: Identity Concealment Response | “I was like, okay, well I’m going to try [hiding my sexuality] because I want to get clean and I’ve had spells where it’s like why? I’m wasting money on drugs. So, let’s go ahead and get clean. I had the first bad experience [of being outed and ridiculed on Facebook] but I was like okay. Maybe if I hide it for as long as I can, I can get some kind of treatment for a while and maybe, maybe I won’t relapse.” (*cisgender man, age 33, Black, bisexual*)  “[The treatment program] wasn’t a lot of people that was LGBT in there so it was kind of, couldn’t really be myself. Yeah, that’s why I didn’t really stay there that long, a couple months…[the staff] would never say…anything [discriminatory], but it was the energy…Just cause like you would go wait in the waiting room area and you would see these brochures. And you would not see one LGBT anything on there and I’m like, wow, like it was just, it was just the energy. It wasn’t, it wasn’t a fit for me.” (*cisgender woman, age 25, multiracial, lesbian*) |
| Support from Staff: Staff as Advocates for LGBTQ+ Clients in Otherwise Stigmatizing Environments | “I’ve had one nurse…[who] asked me if there was anything she could do to make me more comfortable. I asked her if she could paint my fingernails and my toenails. I asked her if she could sit there and watch a movie with me. And little things like that they – she was really, really nice, respectful and she explained to the other nurses and doctors that I was bi, openly bisexual. And she was really kind and caring about it….She’s like, ‘Do you mind if I tell them your sexual orientation?’ I said, ‘Yeah’. She was…just like, ‘Well he’s openly bisexual. You can call him ‘he’. You can say, ‘miss, mister’ as long as you’re nice about it. And after she said that, the nurses who had just come in, ‘How are you feeling today? Are you okay, sir, ma’am?’ Or, they would ask me, you know, ‘What do I want to be called?’” (*cisgender man, age 33, Black, bisexual*) |
| Support from Staff: LGBTQ-Identifying and Allied Providers | “There was some type of…prejudice [from my counselor and peers]…I could hear people make comments. I don’t know if it was just in my head…[my counselor] wasn’t paying attention to me like she did the rest. So that’s why I switched my counselor, actually. It was the only time that I felt, you know, not comfortable being there…the next counselor was transgender. And even though I don’t identify as that…[she] definitely understood me. And she definitely helped me get through when…just gave me some coping skills to deal with [SOGI-related discrimination] and not to take it any offense. You know, she was just a great counselor and really helpful.” (*transgender woman, age 25, Latinx, bisexual*)  “I just talked to [my counselor] a couple of times about my relationship, um, you know, with a female, but at the same time I was talking to another dude as well and she was very supportive. She understood exactly what I meant. She didn’t judge me for being bisexual. She didn’t judge me for having a girlfriend and then talking to a dude.” (*non-binary, age 31, multiracial, bisexual*)  “I mean they’ve just helped me so much and my counselor is actually gay too. So she like understands. We talk, yeah, we talk about all that both issues. Like she really understands you know.” (*cisgender woman, age 26, white, lesbian*)  “… I would just say, you know, [my therapist] makes a, like in the beginning, ‘What do I want to be called,’…just sensitive around certain topics. She wouldn’t act, she would make sure it was comfortable with me before she even asked a question related about anything. She was very sensitive about that, yeah.” (*transgender man, age 23, Black, prefer not to specify sexual orientation*)  “I think I really like my therapist right now. But I’ve been in and out of therapy. That’s probably the first therapist that I could stick with. And he can tell things about me before I even say them; but he obviously waits for me to say them because he wants to hear it from me which I appreciate that level of attention where I know that he’s paying attention to me and picking up on things and kind of knows my patterns of whatever I’m up to…There’s been a lot of work done with dealing with my parents around my gender identity. And I feel like he doesn’t judge me for my drug use. He doesn’t force me to quit. Because in the past I’ve had therapists who are like if you’re using, I’m not going to see you …And I also really appreciate that…he doesn’t push the total sobriety in anything on me.” (*transgender man, age 28, Latinx, gay*)  My therapist is a gay man so he’s of LGBT community, so you know, he’s really sensitive to what he says and you know, he – I’m sure he took training on proper, you know, how to speak and ask certain questions. He doesn’t dig that deep in yet because - like I told him, I’m kind of not ready to get off drugs, but I’m still – that’s why I’m there because I’m just trying. So he knows exactly my own treatment plan and what I want.” (*cisgender woman, age 31, Black, lesbian*) |
| **Substance Use Treatment and Services Experiences: Organizational Policies and Structures (Organizational-Level)** | |
| Discrimination & Stigma in Organizational Structures: Gendered Program Structures | “I’ve considered some inpatient programs. You know, even in healthcare…it’s very much this like binary thing, you know, where it’s like they’ll only accept men patients or women patients or something like that… what if, like I don’t identify as either of those. You know? To find somewhere that would be willing to do that and not like box me in, you know, to one or the other. Because I just don’t fit into those...” (*non-binary and transgender, age 29, Black, queer*)  “I mean the thing within in-patient is it’s so gender divided in a lot of ways because of the roommate situations and things like that…just after I left, the detox I was at split itself two floors into men’s and women’s right after I got out. So it’s a very gendered environment when you’re in an in-patient setting too…so that feels weird because you’re not really sure where you fit in and stuff.” (*transgender man, age 28, Latinx, gay*)  “[The staff] didn’t really know how to go about it because some people felt some type of way cause I use the male bathroom, but ‘She was born a female’, and it’s like that’s not [how I identify]…There’s like three of us, and, um, we all just end up using [the counselor’s] bathroom.” (*transgender man, age 23, Black, prefer not to specify sexual orientation*) |
| Support in Organizational Structures: Gender-Affirming Program Structures | “…this isn’t really like a service, but just to show how much they respected me, they gave me a single room, which was a huge deal. At that point I wasn’t comfortable rooming with a cisgendered man. I wasn’t comfortable rooming with a woman, so they gave me a single room… I feel like having my own room and having that privacy and safety was really important, as well as people at my most recent detox, they put my chosen name as opposed to my legal name on all the forms and on my door. They all called me it and called me ‘he’.” (*transgender man, age 23, white, queer*) |
| Support in Organizational Structures: Affirming Treatment Environment | “You know, they even have drawings that people did about sexuality. They had sayings like all around the center. I would say things like that are attractive and they have actually, you know, a lot of rainbow stuff and it is very welcoming.” (*transgender woman, age 25, Latinx, bisexual*)  “[The program was] actually LGBT friendly like as far as the setting, yeah…they had signs and flags.” (*cisgender woman, age 30, Latinx, pansexual*)  “[The program had] like posters of the same sex. Posters with LGBT referring to like, you know, drugs and treatment. Gives motivational like speeches on the posters… and rainbow flags are definitely a good thing for the LGBT [community]…it was about acknowledgement” (*cisgender woman, age 25, multiracial, lesbian*)  “I used to do testosterone injections. I had a mix of - it was partly going to dispose of my testosterone syringes and also to dispose of my IV drug use syringes. I honestly just went in [to the syringe exchange], gave them an estimate of how many were in my container…That’s why I liked it so much, there was no name exchange. Totally confidential… there were all kinds of people there. Even the person working was visibly trans and that made me feel more comfortable…they didn’t ask any questions about me or who I had sex with or who I was attracted to or been in contact with. They just took the needles and called it good.” (*transgender man, age 23, white, queer*)  “[The harm reduction group is] completely validating of my identity and everything like that…Just knowing it’s there and knowing it’s inclusive…And just they’ve all just always been so supportive. And every time I relapse and every time I do this and that, they’re just always right there.” (*non-binary and transgender, age 24, white, queer*) |
| **Recommendations for LGBTQ+-Affirming SU Treatment and Services** | |
| – Policies | “I think certain [non-discrimination] policies…should be in place so people would know the consequences of their actions before they even say something discriminatory toward LGBTQ+ clients], so maybe somebody won’t say it.” (*cisgender woman, age 31, Black, lesbian*)  “[Non-discrimination policies are] a good thing to have. Because then [LGBTQ+] people are at least protected if something happens.” (*transgender man, age 38, Latinx, queer*)  “I would say [that policies should include] a higher level of discipline for people or clients put other [LGBTQ+] clients in danger.” (*transgender man, age 23, white, queer*)  “If anybody was like treated bad with any discrimination…from a counselor, I think the counselor should be…penalized or maybe sent to…a training on…how to properly talk to a client… I felt like even when I did complain to a director, like no action was done…I feel [there] should be some type of policy about that.” (*cisgender woman, age 30, Latinx, pansexual*)  “I presented the idea of just having everyone — because we would go around anyway at the beginning of group to say our names and give a check in. So like why not add pronouns to that? I think that that would help [to reduce stigma toward transgender and non-binary clients]. Just normalizing that.” (*transgender man, age 28, Latinx, gay*).  “… And I know for certain things you got to say, oh, was you born a male? …But that makes a lot of people, especially that’s trans or on transitioning very, very uncomfortable. I think more policies or procedures on how to like kind of go around that and advocate more towards the client.” (*non-binary, age 31, multiracial, bisexual*)  “… I think it could be helpful to even have LGBT people meeting with people who work at [the office that oversees SUD treatment programs], meeting with directors of these places to give…input to them so that hopefully they can institute better policies or better vetting processes for the staff. I think would be helpful if, I guess, we had more of a voice in how some of these policies are made.” (*cisgender man, age 29, white ,gay*) |
| LGBTQ-specific services | “I think that it could work either way [with LGBTQ-specific or non-specific SU services]. I think it’s good to have a mix of both queer and cis people, so that it really takes away the stigma that any of us are different from each other in that way. Also, I understand the importance of having a queer focus base so that people aren’t in danger of prejudice and even violence towards them because of their identity. You never know how someone is going to react based on their traditions and values. It could go either way. I feel the best-case scenario, there are both options [of LGBTQ-specific and integrated services].” (*transgender man, age 23, white, queer*) |
| Staff Hiring | “I believe as far as counselors, inpatient, outpatient, I think it could be really helpful to have like counselors that identify as LGBT because I think that even though certain counselors are maybe educated in it, it’s different to have someone who identifies that way, maybe has a different perspective because of that. So, I think having a counselor of LGBT could be really helpful.” (*cisgender man, age 29, white*)  “[Onboarding processes] would be used to sort of weed out the people …eliminate the people who aren’t really there for LGBT people instead of people who are just talking to talk, people who also walk the walk.” (*transgender man, age 21, white*) |
| Intake Forms & Processes | “… I think intake forms should not have boxes to check. I think they should have lines to write on… If like, if it’s a health check, and you’re like, do you have asthma? Do you have blood clots? Do you have this? But I mean, if you’re looking for like gender or sexual orientation…When I was at [PROGRAM NAME] this last time, sexual orientation was not a box. It was not gay, straight, bi, whatever. It was like, here’s a line you can write on… And I’m sure like normal straight people are like why is this like this? But like, who cares?...and I understand it’s harder for data collection. But like, I don’t know, someone else can figure out how to make that work… the more like you can let people self-identify, self-disclose, actually the more, better information you get.” (*transgender man, age 38, Latinx, queer*)  “…as far as when they say like female/male, like some people don’t identify as any of those…like some people don’t feel comfortable. And I feel like people are forced and you know, it just makes the [intake] process just a little bit harder when you’re even trying to adapt to a new environment like that and you know, you see something like that, and now you are like wow, this program is going to be like this.” (*cisgender woman, age 30, Latinx, pansexual*)  “I mean, I’m a little undecided about [including sexual orientation and gender identity questions at intake]…I think it is a positive thing because if a person at intake says, ‘Yes, okay, I identify as LGBT,’ then the treatment team can come together and say, okay, maybe it would help to develop their treatment plan as such, include this in a certain way. So, it makes them aware that there might be certain issues there, but then again, there are certain people who are not going to say flat out that they identify as one of those…in that case, if they’re not going to identify as [LGBT], then they can’t get that kind of help…I think it does help to [include those questions].” (*cisgender man, age 29, white, gay*) |
| Provision of Affirming Treatment | “[Treatment discussions should include questions like] ‘…is there anything we need to know? Or is there anything that you’d like to be addressed by in a certain situation? You know, is there a certain trigger?... like asking what sets you off you know…what invalidates you?” (*non-binary and transgender, age 24, white, queer*)  “… [Providers should ask about] where does your family come from…how well do you identify with your family, is it okay to ask about your sexual orientation …Certain questions to give more feedback for the client to make them feel more comfortable and you actually know more too about your client as well in that process.” (*non-binary, age 35, Black, pansexual*)  “Even if [clients] look just like a normal white straight man…they should be asked, you know, how do you identify? Would you like to, how would you like to be addressed?” *(non-binary and transgender, age 24, white, queer*) |
| Staff Training | “… the woman who takes our vitals in detox. And she’s talking about Ellen DeGeneres and was like, ‘She calls herself a husband’. And…didn’t understand the right language to use around Ellen DeGeneres being a lesbian not a trans person. That was really uncomfortable. So I think having some understanding around gender, sexuality and the appropriate things to — you can ask someone their pronouns but don’t ask if they’ve had surgery or something. Things like that. Like, where the line gets crossed and stuff like that.” (*transgender man, age 28, Latinx, gay*)  “I think having the language skills – understanding questions to ask that are pertinent versus questions that are not going to get you anywhere or kind of make you put your shell on... What has made me feel comfortable in the past is… one of my favorite therapists…understood what questions to ask and what to kind of stay away from. For a bisexual woman to have a male provider, even if it’s innocuous, the questions can get creepy fast. Just knowing what my limits are about what I’m willing to talk to you about and what details I’m willing to go into. Not trying to push it. I had one person that reminds me of Mr. Smithers from the Simpson’s, ‘ooh, tell me more.’” (*cisgender woman, age 36, white, bisexual*)  “… I would love for everyone to know that sexual orientation and gender identity are not the same thing. That’s the thing that gets mixed up a lot. Like I get a lot of people say to me, like, well, if you’re into dudes, why didn’t you just stay female? Well, because those two things have nothing to do with each other.” (*transgender man, age 38, Latinx, queer*)  “…the most important thing besides knowing names and pronouns, is knowing how to get the information on how to handle other people responding to the queer person. It’s different for every person. Some people really don’t want others to correct for them. Some people would prefer [others intervene] as opposed to correcting them themselves. Really knowing how to approach that and ask what the person wants without alienating them and making them feel like a burden.” (*transgender man, age 23, white, queer*)  “A certain level of sensitivity [training would be necessary]. [Staff are] walking on eggshells. I feel like I kind of run into that, where it’s almost like they’re trying not to…really address [SOGI], just for fear of…don’t know, maybe being offensive or something like that. But that’s in and of itself what’s hurtful.” (*non-binary and transgender, age 29, Black, queer*)  “I think staff should have trainings on culture because even though if somebody is of LGBT descent, like my experience from being Black is definitely different than somebody being white or even Hispanic or even Asian.” (*cisgender woman, age 31, Black, lesbian*) |
| Visual Cues of an Affirming Treatment Environment | *“…*I think to get out that phobia and not be scared [to discuss SOGI] and make [programs] more welcom[ing] by, I don’t know, signs and banners… You could even put a rainbow in front of your organization like without even saying anything.” (*non-binary, age 35, Black, pansexual*)  “… Some posters, some things like maybe put up a little, um, video when you’re in…the office waiting area, something LGBT-friendly, maybe a little flag. I’m not saying that the whole place on gay pride, but you know, maybe like a little flag…on the side, something that’s welcoming.” (*non-binary, age 31, multiracial, bisexual*)  “I would say like maybe have a room, you know, like a little gallery with information, LGBT groups, you know, talk about the suicide rates, talk about a trans woman getting killed, talk about things like that, have a news article, just like, you know, people get more informed and not even people of LGBT like heterosexuals as well. Everybody, I think, needs to get informed about the, the news or what’s going on.” (*transgender woman, age 25, Latinx, bisexual*) |
| Gender-Affirming Program Structures | “I would say less of the gender segregation and gender treatment…Even though people feel weird about me being a male, at least I know I have that binary to fit into. I can’t imagine to be gender queer and have to go into sober living because you have to pick a side. I would say more queer focused treatment where it’s everyone is invited. Everyone of any gender is able to live there. They always say they are not going to mix genders because of sex, but that’s assuming everyone is straight…I think single stall gender neutral bathrooms are so important. One, there are people that wouldn’t know what bathroom to go into. Two, I’m in this weird place where I’m too feminine looking to go into a male bathroom without getting gazes and I’m too masculine looking to go into female bathrooms without getting gazes.” (*transgender man, age 23, white, queer*) |

*NOTES: AA=Alcoholics Anonymous; LGBTQ+=* *Lesbian, gay, bisexual, transgender, queer, and other populations within the LGBTQ community (e.g., asexual individuals); NA=Narcotics Anonymous; SOGI=sexual orientation and gender identity; SU=substance use*
